# Study of the Relationship between Sigma Receptor Expression Levels and Some Common Sigma Ligand Activity in Cancer Using Human Cancer Cell Lines of the NCI-60 Cell Line Panel

**DOI:** 10.3390/biomedicines9010038

**Published:** 2021-01-05

**Authors:** Evangelia Sereti, Chrisiida Tsimplouli, Elisavet Kalaitsidou, Nikos Sakellaridis, Konstantinos Dimas

**Affiliations:** 1 Department of Pharmacology, Faculty of Medicine, University of Thessaly, 41500 Larissa, Greece; evangelia.sereti@med.lu.se (E.S.); xrisida@med.uth.gr (C.T.); e.kalaitsidou@students.uu.nl (E.K.); nsakella@uth.gr (N.S.); 2 Department of Translational Medicine, Division of Urological Cancers, Lund University, SE 205 02 Malmö, Sweden

**Keywords:** sigma receptors, sigma ligands, cancer, SIGMAR1, PGRMC1, TMEM97, NCI60 COMPARE analysis

## Abstract

Sigma (σ) receptors have attracted great interest since they are implicated in various cellular functions and biological processes and diseases, including various types of cancer. The receptor family consists of two subtypes: sigma-1 (σ1) and sigma-2 (σ2). Both σ receptor subtypes have been proposed as therapeutic targets for various types of cancers, and many studies have provided evidence that their selective ligands (agonists and antagonists) exhibit antiproliferative and cytotoxic activity. Still, the precise mechanism of action of both σ receptors and their ligands remains unclear and needs to be elucidated. In this study, we aimed to simultaneously determine the expression levels of both σ receptor subtypes in several human cancer cell lines. Additionally, we investigated the in vitro antiproliferative activity of some widely used σ1 and σ2 ligands against those cell lines to study the relationship between σ receptor expression levels and σ ligand activity. Finally, we ran the NCI60 COMPARE algorithm to further elucidate the cytotoxic mechanism of action of the selected σ ligands studied herein.

## 1. Introduction

Sigma receptor is a class of receptors that have been the subject of intensive pharmacological research since their discovery in 1976 by Martin et al. [1]. Since then, considerable progress has been made, especially in the last years. It is now known that the family consists of two members: the sigma-1 (σ1) and sigma-2 (σ2) receptors, which are substantially different from each other. Sigma-1 has been cloned by two different groups independently [2,3] and is now widely accepted as a membrane receptor and a chaperone protein with multiple cellular functions [4]. Until recently, the identification of sigma-2 receptor was a matter of debate. In 2011, it was proposed that the PGRMC1 may harbor binding sites for sigma ligands [5]. A few years later in 2017, Alon et al. published evidence showing that the sigma-2 receptor is in fact identical to TMEM97 [6], which is now the prevailing theory. Sigma-2/TMEM97, a member of the insulin-like growth factor binding proteins, is a protein that is reported to be increased in several types of cancer [7]. To complicate things even more, a 2018 study reported that σ2/TMEM97 forms a trimeric complex with PGRMC1 and the low-density lipoprotein receptor (LDLR), which is responsible for the rapid internalization of the low-density lipoprotein (LDL) in HeLa cells, a human cervical cancer cell line [8]. That work suggested that both PGRMC1 and TMEM97 could in fact be part of a more complex receptor that can bind σ2-ligands.

Interestingly, both sigma subtypes have been proposed as therapeutic targets for several diseases and pathological conditions, with various types of cancer being amongst them [9]. However, the precise mechanism of their action is still a matter of debate, even though many independent studies have provided evidence of antiproliferative and cytotoxic activity for both the sigma-1 and sigma-2 receptor ligands [10].

In this study, we try to shed some light on the relationship of the σ1 and σ2 receptors and the anticancer activity of sigma ligands. To this aim, we used Western blot (WB) to study the expression levels of σ1, PGRMC1, and σ2/TMEM97 receptors in several human cancer cell lines, representing nine different cancer types. Most of the tested cell lines included in this study are also included in the NCI60 cell line panel list that is widely used for the in vitro testing of potential anticancer drugs [11]. In addition to the WB studies, we further addressed the in vitro antiproliferative activity of some commonly used σ1 and σ2 ligands against those cell lines to (a) test the efficacy of these ligands against various human cancer types, (b) compare their activity under the same experimental conditions, and (c) assess if there whether any correlation of their activity in relation to the expression of the receptors.

## 2. Materials and Methods

### 2.1. Cell Culture

All cell lines used were purchased from the National Cancer Institute (National Institutes of Health, Bethesda, MD, USA), and were cultured in RPMI 1640 (Roswell Park Memorial Institute 1640) medium (Cat. No. 31870025; Thermo Fisher Scientific, Waltham, MA, USA) supplemented with 5% fetal bovine serum (Cat. No. 1001G; Biosera, Nuaille, France), 2 mM L-glutamine (Cat. No. XC-T1715; Biosera, Nuaille, France), and 100 U/mL penicillin and 100 μg/mL streptomycin (Cat. No. XC-A4122; Biosera, Nuaille, France). Cell cultures were maintained at 37 °C in a humidified incubator with a 5% CO_2_ air atmosphere.

### 2.2. Western Blot Analysis

Cell lysis was performed in T-PER tissue protein extraction reagent (Cat. No. 78510; Thermo Fisher Scientific, Waltham, MA, USA) supplemented with 1% protease and phosphatase inhibitor cocktail (Cat. No. 5872; Cell Signaling Technology, Beverly, MA, USA). Cell lysates were then sonicated (3 cycles, 10 s/cycle) and centrifuged at 13,000 rpm for 30 min at 4 °C, and supernatants were then collected. Protein concentration was determined using a Pierce BCA Protein Assay Kit (Cat. No. 23227; Thermo Fisher Scientific, Waltham, MA, USA). Lysates containing equal amount of protein (20 μg) were run on a 10% acrylamide gel and transferred to a 0.22 μm pore size polyvinylidene difluoride (PVDF) membrane, Immobilon-P^SQ^ Transfer Membrane (Cat. No. ISEQ85R; Merck, Millipore, Darmstadt, Germany). Membranes were blocked for 1 h at room temperature with the appropriate blocking buffer, according to each antibody’s specific protocol, and then incubated overnight at 4 °C with rabbit anti-SIGMA1 antibody (Cat. No. 61994; Cell Signaling Technology, Beverly, MA, USA), rabbit anti-PGRMC1 antibody (Cat. No. 13856; Cell Signaling Technology, Beverly, MA, USA), or rabbit anti-TMEM97 antibody (Cat. No. NBP1-30436; Novus Biologicals, Centennial CO, USA). All antibodies were used at a 1:1000 dilution. Finally, membranes were incubated with the secondary horseradish peroxidase-conjugated anti-rabbit antibody (Cat. No. 7074; Cell Signaling Technology, Beverly, MA, USA) at 1/6000 dilution, developed with Clarity Western enhanced chemiluminescence (ECL) Substrate (Cat. No. 170-5060; Bio-Rad Laboratories, Hercules, CA, USA) and detected with Uvitec Cambridge chemiluminescence imaging system with the help of Alliance Software (ver. 16.06) (Uvitec Cambridge, Cambridge, UK).

### 2.3. Cell Viability Assay

The cytotoxic effect of the sigma ligands Siramesine (kindly offered by H. Lundbeck A/S), PB28 dihydrochloride (Cat. No. sc-204834; Santa Cruz Biotechnology, Dallas, TX, USA), rimcazole dihydrochloride (Cat. No. 1497; Tocris, Abingdon, United Kingdom), BD1047 dihydrobromide (Cat. No. 0956; Tocris, Abingdon, United Kingdom), and SM-21 maleate (Cat. No. sc-204289; Santa Cruz Biotechnology, Dallas, TX, USA) was determined using sulforhodamine B (SRB) colorimetric assay. Cell viability was determined at the beginning of each experiment by trypan blue dye exclusion method and was always greater than 97%. Cells were seeded into a flat bottom 96-well plate at a density of 5000 cells per well, 24 h before treatment. Sigma ligands were dissolved in dimethyl sulfoxide (DMSO) and were serially diluted in culture medium to acquire the desired concentrations. The final concentration of DMSO in each cell culture was no higher than 0.1%. After 48 h treatment, cells were fixed by gentle addition of 50% (*v*/*v*) trichloroacetic acid (TCA) (Cat. No. A1431; Applichem, Darmstadt, Germany) to each well and stained for 1 h at 4 °C. Cells were washed (three times) with slow running tap water; excess water was removed by gentle tapping onto a paper towel and the plates were allowed to air-dry at room temperature (RT). A solution of 0.04% (*w*/*v*) SRB (Cat. No. S9012; Sigma-Aldrich, St Louis, MO, USA) in 1% (*v*/*v*) acetic acid (Cat. No. 45731; Fluka, Buchs, Switzerland) was subsequently used to dye cells for 10 min at RT. After incubation, the excess dye was removed by repeated rinses with 1% (*v*/*v*) acetic acid (Cat. No. 45731; Fluka, Buchs, Switzerland) and cell monolayers were allowed to dry at RT. The protein-bound dye was dissolved in 10 mM Tris base solution by incubation for 10 min at 37 °C followed by solubilization in an orbital shaker for 10 min. Absorbance was measured at 540 nm in a BioTek EL311 microplate reader (BioTek Instruments, Winooski, VT, USA). From the generated dose-response curves, we determined the GI_50_ (50% cell growth inhibition), TGI (total growth inhibition), and LC_50_ (50% lethal concentration) parameters. Growth inhibition of 50% (GI_50_) is calculated from ((Ti-Tz)/(C-Tz)) x 100 = 50. The drug concentration resulting in total growth inhibition (TGI) is calculated from Ti = Tz. The LC_50_ indicating a net loss of cells following treatment is calculated from ((Ti-Tz)/Tz) x 100 = − 50. In these formulas Tz represents a measurement of the cell population for each cell line at the time of drug addition, Ti is a measurement representing the growth of cells in the presence of a given concentration of the drug and C the measurement of the control cells at the end of the 48 h incubation period.

Following standardization, the GI_50_ z-scores were defined and cell lines were categorized, with z-scores ≥ 0.8 categorized as being resistant and z-scores ≤ 0.8 as being sensitive to the compounds tested.

### 2.4. Statistical Analysis

All data are presented as the mean ± SD of at least 3 independent experiments. A two-tailed Student’s *t*-test was used to calculate the statistical significance between sigma receptors’ expression levels in the cancer cell lines tested versus cell lines with the lowest protein expression levels. A two-way ANOVA with either Bonferroni’s or Tukey’s post hoc test was used to determine statistical significance of sigma ligands’ antiproliferative activity within cell lines of the same cancer type. Data normalization was performed by z-score analysis. Pearson’s linear correlation coefficient (r) was calculated to measure the strength of the linear relationship between the antiproliferative efficacy of the selected sigma ligands to the expression levels of sigma receptors. The NCI60 COMPARE algorithm was used to calculate the Pearson correlation coefficient (PCC) of the sigma ligands against compounds with known mechanism of action (MoA) in the National Cancer Institute (NCI) database. Differences were considered significant (rejection of the null hypothesis) when *p* ≤ 0.05.

## 3. Results

### 3.1. σ1 Receptor, PGRMC1, and σ2/TMEM97 Were Heterogeneously Expressed in 23 Human Cancer Cell Lines

**Figure 1:**
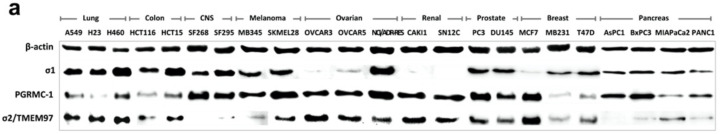

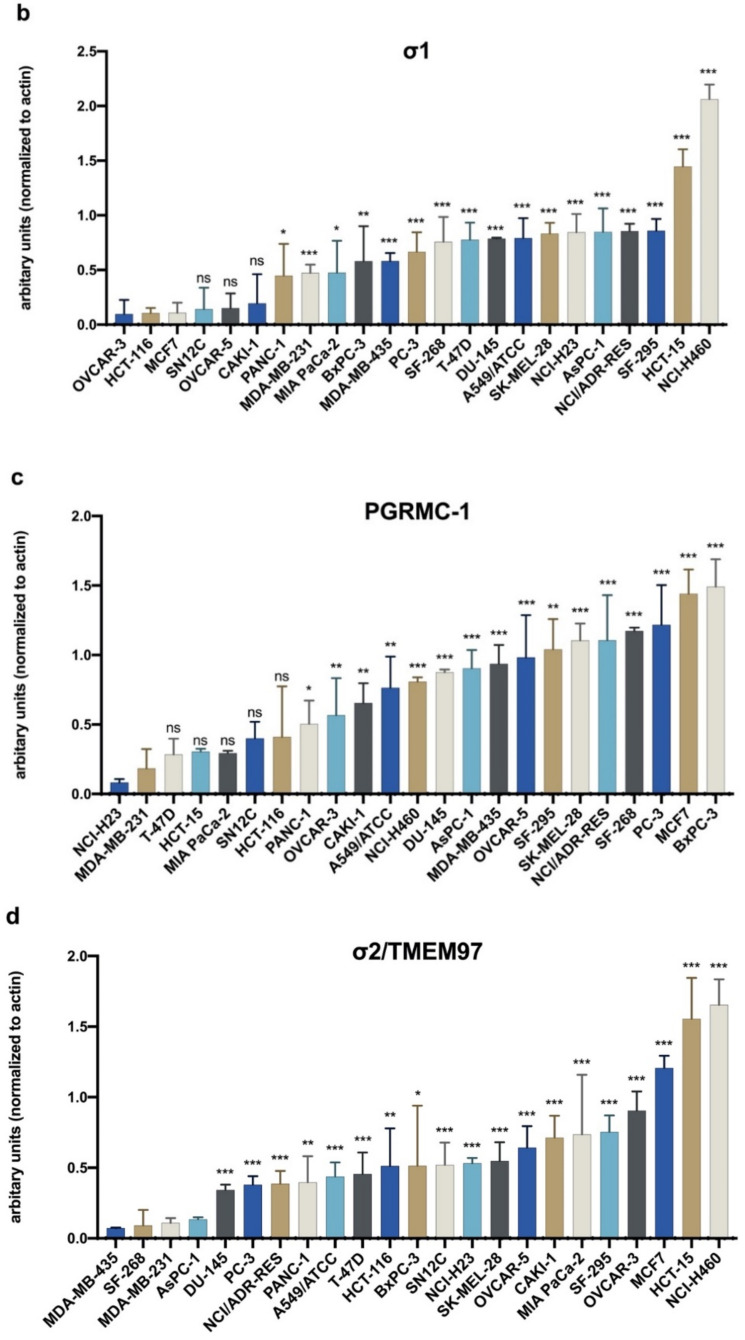
Expression levels of σ1, PGRMC1, and σ2/TMEM97 in the 23 human cancer cell lines. (**a**) Western blot analysis of σ1, PGRMC1, and σ2/TMEM97 expression in 23 human cancer cell lines representing nine different cancer types. β-Actin was used as a loading control. Bar graphs of σ1 (**b**), PGRMC1 (**c**), and σ2/TMEM97 (**d**) relative expression levels normalized to β-actin. Results are expressed as mean ± SD of at least three independent experiments. Statistical significance was calculated versus cell lines with the lowest protein expression levels: OVCAR-3, HCT-116, MCF-7 (**b**); NCI-H23, MDA-MB-231 (**c**); and MDA-MB-435, SF-268, MDA-MB-231, AsPC-1 (**d**). * *p* ≤ 0.05; ** *p* ≤ 0.01; *** *p* ≤ 0.001; ns: not significant.

For the first time in the literature, the expression levels of σ1 receptor, PGRMC1, and σ2/TMEM97 were studied simultaneously by Western blot analysis in 23 human cancer cell lines. The cell lines used represent nine different types of cancer: lung, colon, CNS, melanoma, ovarian, breast, renal, prostate, and pancreatic ductal adenocarcinoma (PDAC). Figure 1a shows that σ1, PGRMC1, and σ2/TMEM97 receptors were expressed with different expression patterns in the studied cell lines. Sigma-1 receptor showed the highest expression levels in NCI-H460 (lung cancer) and HCT-15 (colon cancer) cell lines, while the lowest expression levels were reported in CAKI-1, SN12C (kidney cancer), OVCAR-3, OVCAR-5 (ovarian cancer), HCT-116 (colorectal cancer), and MCF7 (breast cancer) cell lines (Figure 1b). Sigma-2/TMEM97 receptor was found to be highly expressed in NCI-H460 (lung cancer), HCT-15 (colorectal cancer), and MCF7 (breast cancer) cell lines, with low expression levels in MDA-MB-435 (melanoma), SF268 (CNS cancer), and MDA-MB-231 (breast cancer) cell lines. Regarding PGRMC1 receptor, BxPC-3 (pancreatic cancer) and MCF-7 (breast cancer) cell lines expressed the highest levels, while the lowest levels were observed in NCI-H23 (lung cancer), HCT15, HCT116 (colon cancer), MDA-MB-231 (triple negative breast cancer), and T-47D (breast cancer) cell lines (Figure 1c). Despite the different profiles reported above, it seemed that there were few exceptions where the σ receptors did share similarities in terms of their expression levels. Specifically, in NCI-H460 and HCT-15 cell lines, both σ1 and σ2/TMEM97 showed the highest expression levels among all the cell lines studied. Moreover, MCF7 cell line expressed high levels of both σ2/TMEM97 and PGRMC1 receptors. However, σ1, PGRMC1, and σ2/TMEM97 were generally expressed differentially, even between cell lines of the same cancer type. Examples of this were σ1 expression in ovarian and breast cancer, PGRMC1 expression in lung and breast cancer, and σ2/TMEM97 in melanoma and breast cancer cell lines.

### 3.2. In Vitro Antiproliferative Efficacy of Sigma Ligands

**Figure 2:**
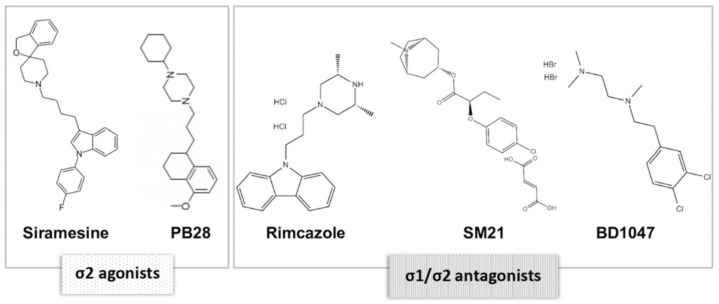
Chemical structures of the selected sigma receptors agonists and antagonists siramesine, PB28 dihydrochloride (PB28), rimcazole, SM21 maleate (SM21), and BD1047 dihydrobromide (BD1047).

The in vitro antiproliferative efficacy of five common sigma ligands was investigated in the same panel of the 23 human established cancer cell lines in which we earlier investigated the expression levels of σ1, PGRMC1, and σ2/TMEM97 receptors. The selected sigma ligands were siramesine and PB28 dihydrochloride, referred to in the literature as σ2 agonists with high σ2 receptor affinities [12,13], and rimcazole [14], SM-21 maleate [15], and BD-1047 dihydrobromide [16], referred to as σ1/σ2 antagonists. The chemical structures of the sigma ligands tested are shown in Figure 2.

**Figure 3:**
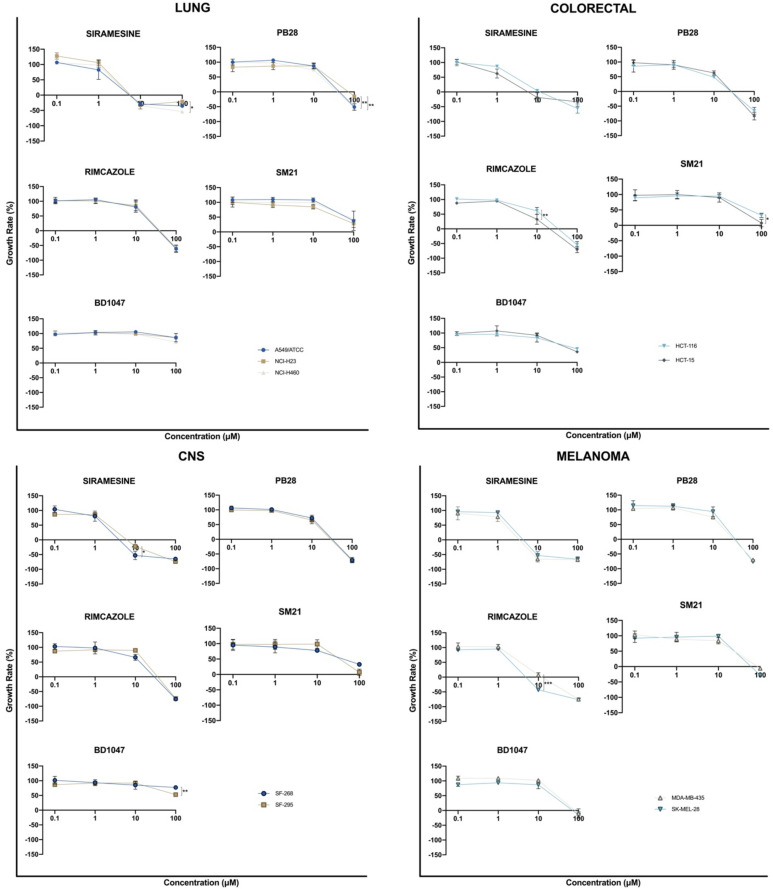

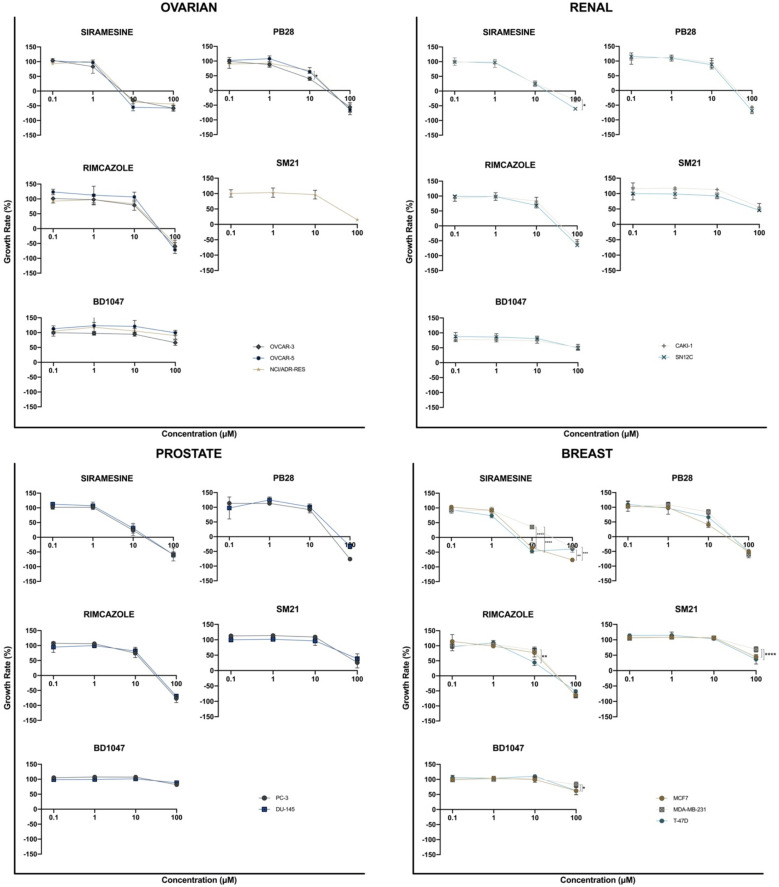

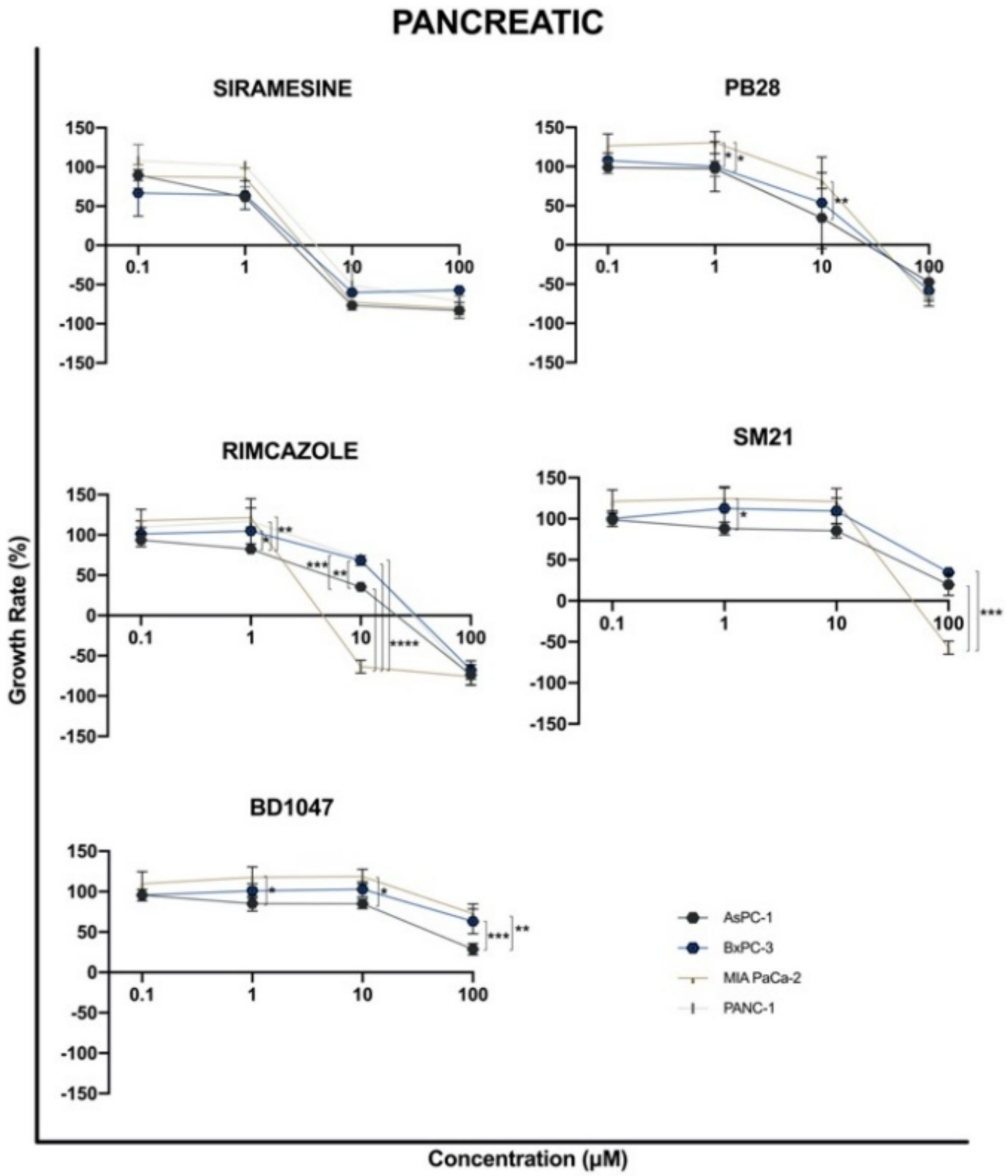
Dose–response effect of the selected sigma ligands siramesine, PB28 dihydrochloride (PB28), rimcazole, SM21 maleate (SM21) and BD1047 dihydrobromide (BD1047) in 23 human cancer cell lines. Cells were exposed to different concentrations of the compounds for 48 h and growth rates were calculated with sulforhodamine B (SRB) assay. All compounds were tested at four concentrations (one log serial dilutions from 10^−4^ M to 10^−7^ M). The results are the average of three independent experiments; each one ran in triplicate. * *p* ≤ 0.05; ** *p* ≤ 0.01; *** *p* ≤ 0.001; **** *p* ≤ 0.0001.

Twenty-three established human cancer cell lines were treated with the sigma ligands for 48 h at various concentrations (100, 10, 1, and 0.1 μΜ), and a cell viability assay (SRB) was performed. Dose–response curves were generated after incubation with different concentrations of the various sigma ligands (Figure 3).

From the dose–response curves, GI_50_, TGI, and LC_50_ parameters were calculated for each cell line, representing the antiproliferative, cytostatic, and cytotoxic effects, respectively. Therapeutic index (TI) was also calculated as LC_50_ to GI_50_ ratio, determining the relative safety of the compounds (Table 1).

Siramesine was found to exhibit the best in vitro antiproliferative activity among the sigma ligands tested, as indicated by the low GI_50_ values in all studied cell lines (mean GI_50_ = 4.3 μΜ). Rimcazole (mean GI_50_ = 22.3 μΜ) and PB28 (mean GI_50_ = 24.6 μΜ) demonstrated moderate antiproliferative efficacy, while SM21 (mean GI_50_ = 73.6 μΜ) and BD1047 (mean GI_50_ = 92.1 μΜ) showed no significant cell growth inhibition efficacy. Siramesine exhibited the best cytostatic and cytotoxic efficacy, as indicated by the low TGI mean parameter of 15.3 μΜ and low LC_50_ mean parameter of 45 μΜ.

**Figure 4:**
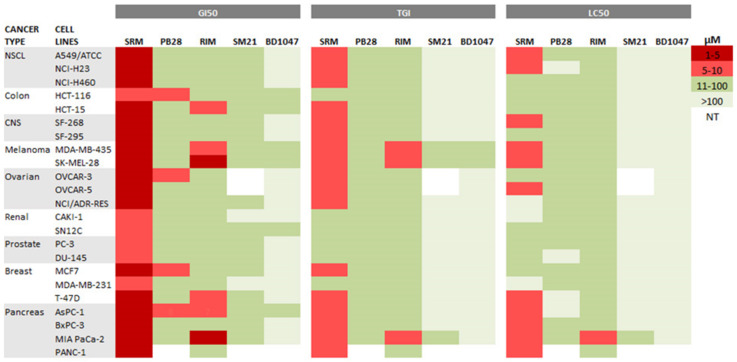
Heat maps representing GI_50_, TGI, and LC_50_ parameters of the sigma ligands tested against 23 human cancer cell lines. A warm-to-cool color spectrum (red > light red > green > light green) is used to represent the parameter value range (1–5 μΜ, 5–10 μΜ, 10–100 μΜ, >100 μΜ), respectively. White color stands for not tested (NT) cell lines. SRM: siramesine; RIM: rimcazole.

GI_50_, TGI, and LC_50_ parameters of the selected sigma ligands against the 23 established human cancer cell lines were further visualized by heat correlation maps (Figure 4). The parameter values were coded on a warm-to-cool color spectrum in which the warmer color (red) corresponds to the lowest values, while the colder color (light green) corresponds to the highest values. Low parameter values indicate better sigma ligands activity. Figure 4 shows that the GI_50_ parameter values for siramesine were in the warm range of the color scale (red, light red) in all the tested cancer cell lines, which contrasted with GI_50_ parameter values for the other tested sigma ligands, which were in the cold color region (green, light green). The same pattern was observed for TGI and LC_50_ parameters, as siramesine was in the warm region of the color scale in several cell lines, in contrast to the rest of the sigma ligands tested. This indicates that among the sigma ligands studied, the σ2 agonist siramesine exhibited the best antiproliferative, cytostatic, and cytotoxic activity. Unlike siramesine, PB28 was found to exhibit significant antiproliferative activity against only four cell lines (HCT116—colorectal cancer, OVCAR3—ovarian cancer, MCF7—breast cancer, and AsPC1—pancreatic cancer). Interestingly, rimcazole’s highest activity was observed against the two melanoma cell lines tested and two out of the four pancreatic cancer cell lines (AsPC1 and MiaPaCa2) tested.

**Figure 5:**
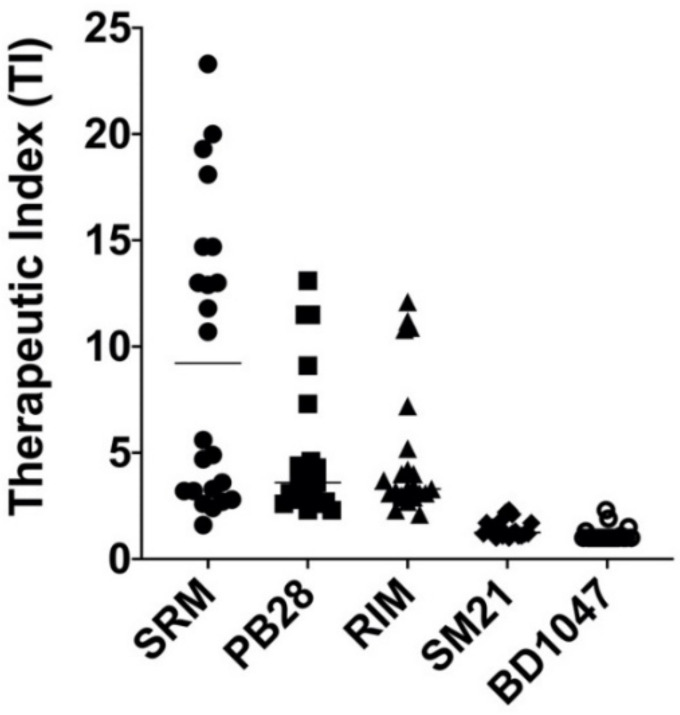
Therapeutic index (TI) values of the selected sigma ligands (SRM, PB28, RIM, SM21, and BD1047) for the 23 human cancer cell lines tested in this study. SRM: siramesine; RIM: rimcazole. Note: In cases where GI_50_ and LC_50_ were higher than the highest tested concentration (100 μM), the TI was calculated using GI_50_ and LC_50_ at 100 μΜ.

The therapeutic index (TI) of the selected ligands was also calculated as an LC_50_ to GI_50_ ratio as an indicator of the relative safety of a substance (Figure 5). High TI values indicate a more favorable safety profile. Figure 5 shows that the σ2 agonist siramesine has a very good therapeutic index (>10) in many of the cell lines studied in comparison to the other sigma ligands tested.

### 3.3. Sigma Ligands’ Sensitivity Was Not Correlated to Their Activity as Either Agonists or Antagonists

**Figure 6:**
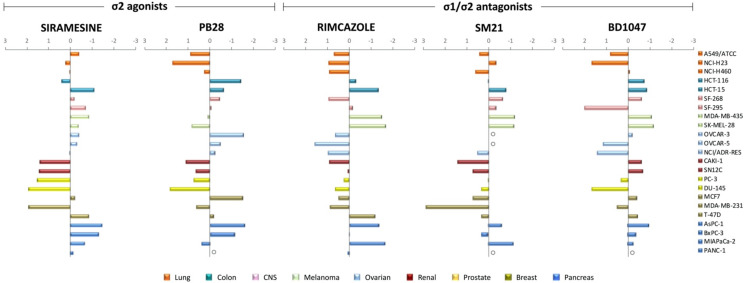
Distribution of z-score-normalized GI_50_ parameter values for each sigma ligand against 23 human cancer cell lines. Cell lines are color-coded to represent different cancer types. The vertical line represents the mean response. Sigma ligand-sensitive cell lines are projected to the right of the vertical line while sigma ligands-resistant cell lines are projected to the left of the vertical line. Open circle (○) means not tested.

We next sought to identify possible patterns in the sigma ligands’ sensitivity profiles that could be related to the sigma ligands activity as either agonists or antagonists. GI_50_ values were normalized using the z-score statistical parameter and grouped by cancer type (Figure 6). The z-score normalization reflects the GI_50_ deviation from the mean, in terms of standard deviation. Positive and negative values were plotted along a vertical line that represents the mean GI_50_ value of all the cell lines against the tested sigma ligand. Negative values, projected to the right, represent cellular sensitivities that exceed the mean (i.e., more sensitive cell lines), while positive values, projected to the left, represent cell lines less sensitive to the mean (i.e., more resistant cell lines). Figure 6 shows that there was no distinct sensitivity pattern of the sigma ligands studied herein, regardless of their receptor specificity or the agonistic/antagonistic classification. Instead, it seemed that there was a general sensitivity/resistance pattern, discrete for each cell line, against the effect of the selected sigma ligands. These distinct responses may reflect a cell line or, in some cases, tissue-dependent sigma ligand effect. Indeed, our data suggest that all the lung, renal, and prostate cancer cell lines tested are resistant against all the studied sigma ligands, whereas all colorectal, melanoma, and pancreatic cancer cell lines appeared to be sensitive against the effect of all sigma ligands studied. Focusing on siramesine, which our data indicates has the best in vitro anticancer efficacy, it appears that pancreatic cancer cell lines are more sensitive against siramesine’s activity, suggesting that siramesine should be further investigated as a novel putative therapeutic approach in the treatment of pancreatic cancer.

### 3.4. Sigma Receptors Expression Levels Did Not Appear to Correlate with the Sigma Ligands’ Antiproliferative Effect

**Figure 7:**
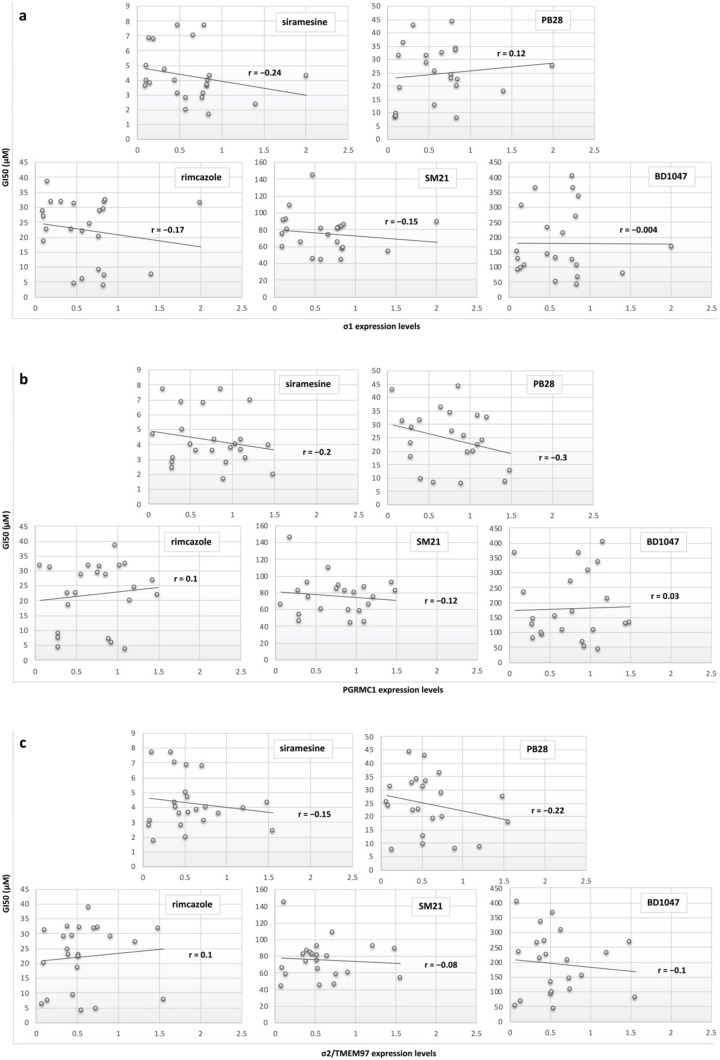
Scatter plots correlating sigma ligand antiproliferative efficacy (GI_50_) versus σ1 expression levels (**a**), PGRMC1 expression levels (**b**), and σ2/TMEM97 expression levels (**c**).

Pearson’s linear correlation coefficient (*r*) was calculated to investigate the relationship between the antiproliferative efficacy of the selected sigma ligands to the expression levels of sigma receptors. The Pearson’s *r* is a statistical measure of the strength of the linear relationship between two variables and is constrained between values −1 ≤ r ≤ 1. Positive *r* values denote positive linear correlation while negative values denote negative linear correlation. An *r* of −1 indicates a perfect negative linear relationship, an *r* of 0 indicates no linear relationship, and an *r* of 1 indicates a perfect positive linear relationship. Scatter plots of the tested sigma ligands’ antiproliferative effect (GI_50_) versus σ1 expression levels (Figure 7a), PGRMC1 expression levels (Figure 7b), and σ2/TMEM97 expression levels (Figure 7c) were generated, and the Pearson’s *r* was calculated for each relationship. Figure 7a shows that there was a weak negative linear correlation (*r* = −0.24) between siramesine antiproliferative effect and σ1 expression levels, while no other significant correlation was reported between the other tested sigma ligand efficacies and the σ1 expression levels. Figure 7b shows that there was a weak negative linear correlation between siramesine and PB28 antiproliferative effect and PGRMC1 expression levels (*r* = −0.2 and *r* = −0.3, respectively), while no other significant correlation was reported between the other tested sigma ligand efficacies and the PGRMC1 expression levels. Figure 7c shows that there was a weak negative linear correlation between PB28 antiproliferative effect and σ2/TMEM97 expression levels (*r* = −0.22), while no other significant correlation was reported between the other tested sigma ligand efficacies and the σ2/TMEM97 expression levels.

### 3.5. Putative Sigma Ligands’ Mechanism of Action: Prediction Using the COMPARE Algorithm

**Figure 8:**
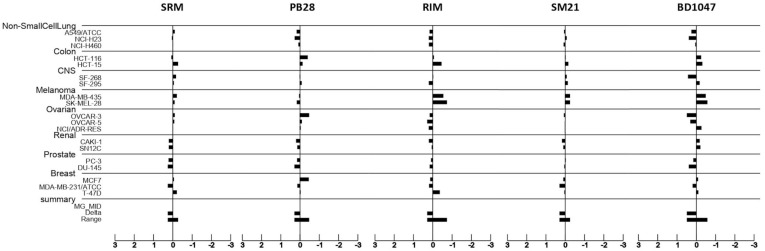
Mean graph plots generated from the National Cancer Institute (NCI, National Institutes of Health, Bethesda, MD, USA) database for the tested sigma ligands siramesine (SRM), PB28 dihydrochloride (PB28), rimcazole (RIM), SM21 maleate (SM21), and BD1047 dihydrobromide (BD1047). Sensitive cell lines are projected to the right of the vertical line, while resistant cell lines are projected to the left of the vertical line. The mean graphs for each of the sigma ligands studied were further used to run the COMPARE algorithm and calculate the Pearson correlation coefficient (PCC) against compounds in the NCI database with known mechanism of action (MoA). The PCC is a measurement of pattern similarity and as such, substances with similar mean graphs show high similarity (high PCC) in their anticancer mechanisms in vitro. The highest similarity between two compounds is expressed with a PCC value approaching 1. A PCC of −1.0 denotes a perfect mirror image, while a PCC of 0 means that there is no correlation between the two compounds [19]. Table 2 shows data retrieved after running the COMPARE algorithm against the NCI standard agents’ database and presents the five substances with the highest correlation to the selected sigma ligands, as proposed by the algorithm.

Sigma ligands’ possible mechanism of action (MoA) was investigated using the COMPARE algorithm. The COMPARE algorithm from the Developmental Therapeutics Program, NCI, was used at the GI_50_ level in order to identify compounds from the standard agent database sharing similar mechanisms of growth inhibition with the sigma ligands studied herein. Pairwise correlation coefficients of greater than 0.6 were used as the cut-off for assessing whether two agents were likely to share a similar MoA [17,18]. The NCI anticancer screen employs 60 human tumor cell lines (NCI60 panel) that have been grouped in cancer type subpanels. Since pancreatic cancer is not included in the NCI60 panel, COMPARE analysis was performed on 19 of the 23 human cancer cell lines included in this study. Dose–response curves for each cell line assessed against the tested sigma ligands were converted into “endpoint” patterns, thus giving a snapshot of sigma ligands’ activity. GI_50_ values for each of the 19 cell lines plotted against the tested sigma ligands were calculated from the dose–response curves (Figure 3) and converted to their log10 GI_50_ values. Log10 GI_50_ values were averaged, and each value was subtracted from the average to create positive and negative values, referred to as deltas. Plotted delta values along a vertical line represent the mean response of each cell lines in the panel to the test agent. The “mean graph” pattern was unique for each sigma ligand (Figure 8). Positive values project to the right of the vertical line and represent cellular sensitivities to the tested sigma ligand that exceed the mean, while negative values project to the left of the vertical line and represent cell line sensitivities to the tested sigma ligand that were less than the average value. Consistent with our previous observations on the z-score-normalized GI_50_ parameter values for each cell line (Figure 6), the mean graph display showed little similarities between the sigma ligands tested.

The highest PCC (0.884) was reported for rimcazole to thalicarpine. Thalicarpine (or thaliblastine) is a natural vinca alkaloid with antineoplastic activity, which induces single strand breaks in DNA and arrests cancer cells at the G2/M and G1 phase of the cell cycle. It is also reported to exhibit RNA- and protein synthesis-inhibiting activities [20]. Interestingly, spirogermanium, the second most related compound to rimcazole (PCC 0.687), is also related to protein synthesis inhibition (Table 2, [4]). The second highest correlation, as determined by the PCC, was PB28 with pibenzimol hydrochloride (PCC 0,724), an agent known to inhibit DNA replication by inhibiting topoisomerase I and DNA polymerase. BD1047 and siramesine showed only weak correlations with the tested agents from the standard agents’ database (Table 2). BD1047 showed a weak correlation (PCC 0.618) with spirogermanium, which, as mentioned above, is a protein synthesis inhibitor and tamoxifen that reduces DNA synthesis and cellular response to estrogen [21]. Finally, siramesine was found to be weakly associated with dihydro-5-azacytidine (PCC 0.601). Dihydro-5-azacytidine is a synthetic nucleoside analogue of deoxycytidine that inhibits DNA methyltransferase, thereby interfering with abnormal DNA methylation patterns that are associated with genetic instability in some tumor cells consequently acting as an epigenetic modifier drug.

## 4. Discussion

Sigma receptors have attracted increased research interest over the last few decades, as reflected in the growing number of extensive and complex literature published in this rapidly evolving field. It is now undisputed that σ1 receptor is an allosteric modulator of multiple cellular signaling systems that, in the context of cancer, act as a chaperone protein, which is a component of the cancer cell support machinery [27]. Until recently, σ2 receptor was considered to be related to the progesterone receptor membrane component 1 (PGRMC1) [5]. More recently, studies have revealed the molecular identification of σ2 receptor as TMEM97 [6], thereby elucidating a longstanding pharmacological mystery and paving the way to applying modern molecular biology tools and techniques to mechanistic studies of this receptor. However, the function of σ2 receptor remains a mystery and to make things more complex, recent studies show that PGRMC1 and TMEM97 may co-operate and form a ternary complex of LDLR–PGRMC1–TMEM97 that results in the rapid internalization of LDL by LDLR in HeLa cells [8].

Several studies report overexpression of σ receptors in cancer; however, these studies mainly involved the indirect detection of σ receptors by radio-binding of their selectively labeled ligands [28,29,30,31]. In this study, we determined for the first time the expression levels of σ receptors by direct detection of their protein expression via Western blotting, investigating the expression levels of the σ1, σ2/TMEM97, and PGRMC1 receptors simultaneously in 23 human cancer cell lines, representing nine different types of cancer. Our data suggest that σ1, PGRMC1, and σ2/TMEM97 receptors are expressed in the majority of the 23 cell lines tested, exerting different expression patterns without showing any selectivity to a particular cancer type. However, we should emphasize that both σ1 and σ2/TMEM97 showed the highest expression levels in NCI-H460 (non-small cell lung cancer) and HCT-15 (colorectal cancer) cell lines. Of note, several reports suggest overexpression of TMEM97, otherwise known as MAC30, and a correlation with poor outcome in lung and colorectal cancer [32,33,34]. Moreover, even though the expression of these receptors was diverse, the breast cancer cell line MCF7 expressed high levels (as compared to the majority of the rest of the cancer cell lines tested herein) of both σ2/TMEM97 and PGRMC1 receptors. These findings are to an extent in agreement with data in the literature reporting high expression levels of σ1 receptor in lung cancer [35] and high expression levels of σ2 [36] and PGRMC1 [37] in MCF7 breast cancer cell line. Further, in agreement with the work published by Vilner et al. based on radio-ligand assays [36], sigma-1 receptor was found to be expressed at very low levels in the MCF7 cell line. Overall, we here provide novel data regarding expression—especially of PGRMC1 and σ2/TMEM97, for which very little is known [9]—in different human cancer types and cell lines included in a significant tool for anticancer drug development, the NCI60 cell line panel. Additionally, in light of the putative interaction between these two receptors reported previously [8], it seems that their interrelationship is far more complex as this is judged from the differences in their expression levels in the cell lines tested herein.

On the basis of our data showing that σ1, PGRMC1, and σ2/TMEM97 receptors are highly expressed in many of the cell lines tested, we further explored whether sigma receptors mediate the in vitro anticancer activities of five commonly used sigma ligands. In this context, we investigated the in vitro antiproliferative efficacy of siramesine, PB28 dihydrochloride, rimcazole dihydrochloride, BD1047 dihydrobromide, and SM-21 maleate under the same experimental conditions and against the same panel of the 23 human established cancer cell lines in which the expression levels of σ1, PGRMC1, and σ2/TMEM97 receptors were previously studied. The selected sigma ligands were representative sigma agonists and antagonists with high affinities for σ1 and/or σ2 receptor. It is worth noting that in the literature, the classification of sigma ligands as agonists or antagonists is based on the recapitulation of the σ1 gene overexpression or knockdown phenotypes [38]. First, we attempted to identify possible patterns in the sigma ligands’ sensitivity profiles that could be related to the sigma ligands activity as either agonists or antagonists. We must underline that this is the first report where the anticancer activity of those agents is tested under the same experimental conditions and following the guidelines and requirements of the NCI Developmental Therapeutics Program (National Cancer Institute, National Institutes of Health, Bethesda, MD, USA; https://dtp.cancer.gov/), the most successful and comprehensive program worldwide established since 1955 and dedicated to the discovery and development of anticancer drugs. Through this program, many important anticancer drugs such as taxanes, vinca alkaloids, and even more advanced anticancer approaches such as sipeulucel-T have entered clinics. Our data suggest that under the experimental conditions tested in this study, there was no distinct sensitivity pattern between the sigma receptor agonists compared to the sigma receptor antagonists studied. Pearson’s linear correlation coefficient (*r*) was calculated to further investigate the relationship between the antiproliferative efficacy of the selected sigma ligands and the expression levels of sigma receptors. Our data confirmed that there was no significant relationship between sigma receptor expression levels and the sigma ligands’ antiproliferative effect (−0.3 < *r* < 0.22). These results indicate that the compounds tested in this study exert their effect mainly through mechanisms that do not involve the σ receptors, even though they show in vitro anticancer activity. A recent study by the group of Mach et al. reported that sigma-2 receptor/TMEM97 and PGRMC1 do not mediate sigma-2 ligand cytotoxicity [39], supporting this assumption. Among the tested σ ligands, the σ2 agonist siramesine was found to exhibit the best antiproliferative, cytostatic, and cytotoxic activity, and thus appears to be the most promising compound. It is also worth noting that, among the nine cancer types studied, pancreatic cancer appears to be the most sensitive cancer type against siramesine. Moreover, of interest is the finding that whilst rimcazole was, in general, found to exert weak anticancer efficacy, the two melanoma cell lines tested were found to be very sensitive to the activity of this compound, even though the σ1 levels in these cell lines were not the highest amongst the 23 cell lines tested herein.

An essential missing element in sigma receptor biology in cancer is a clear definition of the underlying molecular MoA that translates into the cellular response of the sigma receptor ligand effect. In the literature, many potential mechanisms of action have been proposed to mediate sigma ligands’ anticancer efficacy, including apoptosis [40,41], autophagy [42], lysosomal destabilization [43], mitochondria destabilization [44], and ferroptosis [45,46]. The diversity in the mechanisms of action show that ligand binding of sigma receptors is a multistep process that may be ligand- or cancer type-dependent, thus underlining the significant need to further validate and expand our knowledge in this field. In this context, we attempted to investigate the potential mechanisms of action of the selected sigma ligands using, for the first time in the field, the COMPARE NCI60 anticancer drug screening algorithm. Consistent with our previous observations, the mean graph displays of the NCI60 cell line screening for the selected sigma ligands, showing only minor similarities between the sigma ligands tested. Interestingly, the most common MoA was found to be related to either the synthesis of DNA or the integrity of DNA followed by protein and RNA synthesis inhibition, as judged by the PCC of the sigma ligands and the standard agents returned after running the COMPARE algorithm. It is also important to note that siramesine, which was found to be the most potent agent, appeared to have the highest degree of association with the DNA damage substance dihydro-5-azacytidine, followed by the alkylating agent cyanomorpholino-ADR, although, in general, these are considered to be moderate-to-low associations on the basis of the corresponding PCC. Thus, an interaction of this compound with DNA might be a part of its MoA.

In conclusion, we show that sigma receptors are indeed expressed in the majority of the 23 human cancer cell lines assessed in this study. We provided evidence that sigma receptor expression does not follow a cancer-type pattern or (with some exceptions) a pairwise expression pattern and that it may not mediate the antiproliferative efficacy of a panel of some very common σ ligands studied in this report. Among the ligands tested, the σ2 receptor ligand siramesine showed the most promising anticancer activity, especially against pancreatic cancer. These findings also provide a platform that rationally warrants the further evaluation of siramesine as a potential anticancer compound, especially against pancreatic cancer.

## Figures and Tables

**Table 1:** GI_50_ (50% cell growth inhibition), TGI (total cell growth inhibition), LC_50_ (50% lethal concentration), and therapeutic index (TI) parameters for each cell line and compound tested as calculated by the graphs shown in Figure 3. Dots (•) mean not tested. For more details on the three parameters see M&M under “2.3 Cell viability assay”. Note: In cases where GI_50_ and LC_50_ were higher than the highest tested concentration (100 μM), the TI was calculated using GI_50_ and LC_50_ at 100 μΜ.

CANCER TYPE	CELL LINES	SIRAMESINE				PB28				RIMCAZOLE				SM21				BD1047			
		GI50	TGI	LC50	TI	GI50	TGI	LC50	TI	GI50	TGI	LC50	TI	GI50	TGI	LC50	TI	GI50	TGI	LC50	TI
**NSCL**	A549/ATCC	3.6	7.7	11.7	3.3	34.2	66.8	99.3	2.9	29.2	61.0	92.7	3.2	84.2	100.0	100.0	1.2	>100	>100	>100	1.0
	NCI-H23	4.7	7.9	11.2	2.4	42.9	87.0	>100	2.3	31.9	62.8	93.7	2.9	65.2	100.0	100.0	1.5	>100	>100	>100	1.0
	NCI-H460	4.3	7.7	83.1	19.3	27.5	62.6	97.7	3.6	31.6	63.6	95.6	3.0	88.9	100.0	100.0	1.1	>100	>100	>100	1.0
**Colon**	HCT-116	5.0	15.9	90.5	18.1	9.6	48.0	87.4	9.1	18.5	57.5	96.5	5.2	74.5	100.0	100.0	1.3	89.9	>100	>100	1.1
	HCT-15	2.4	7.9	13.5	5.6	17.9	48.6	79.4	4.4	7.5	38.5	82.1	10.9	53.7	100.0	100.0	1.9	77.8	>100	>100	1.3
**CNS**	SF-268	3.1	6.4	9.8	3.2	24.0	55.5	86.9	3.6	20.1	51.9	83.7	4.2	65.5	100.0	100.0	1.5	>100	>100	>100	1.0
	SF-295	4.0	8.2	58.6	14.7	19.9	52.5	85.0	4.3	31.9	59.4	86.9	2.7	57.7	100.0	100.0	1.7	>100	>100	>100	1.0
**Melanoma**	MDA-MB-435	2.8	5.9	9.0	3.2	25.6	56.6	87.7	3.4	6.0	10.7	72.5	12.1	44.0	94.2	100.0	2.3	51.7	10.1	>100	1.9
	SK-MEL-28	3.6	6.7	0.8	2.7	33.4	59.9	86.3	2.6	3.9	7.2	28.4	7.2	44.6	80.1	100.0	2.2	42.6	86.8	>100	2.3
**Ovarian**	OVCAR-3	3.6	7.6	71.9	20.0	8.2	47.5	94.0	11.5	28.8	61.3	93.8	3.3	•	•	•	•	>100	>100	>100	1.0
	OVCAR-5	3.8	6.7	9.7	2.6	19.5	54.4	89.3	4.6	38.7	64.0	89.2	2.3	•	•	•	•	>100	>100	>100	1.0
	NCI/ADR-RES	4.3	7.6	>100	23.3	22.3	56.3	90.2	4.0	32.4	65.9	99.3	3.1	86.3	100.0	100.0	1.2	>100	>100	>100	1.0
**Kidney**	CAKI-1	6.8	50.7	>100	14.7	36.4	66.5	96.5	2.7	31.8	64.8	97.8	3.1	100.0	100.0	100.0	1.0	>100	>100	>100	1.0
	SN12C	6.9	36.2	89.3	13.0	31.5	60.2	89.0	2.8	22.6	56.3	90.0	4.0	91.9	100.0	100.0	1.1	98.5	>100	>100	1.0
**Prostate**	PC-3	7.0	35.9	90.5	12.9	32.5	59.3	86.0	2.6	24.6	31.2	84.3	3.4	73.9	100.0	100.0	1.4	>100	>100	>100	1.0
	DU-145	7.7	40.6	90.8	11.8	44.3	77.9	>100	2.3	28.8	58.7	88.5	3.1	82.3	100.0	100.0	1.2	>100	>100	>100	1.0
**Breast**	MCF7	4.0	7.5	42.3	10.7	8.6	49.8	98.8	11.5	27.1	58.8	90.5	3.3	91.8	100.0	100.0	1.1	>100	>100	>100	1.0
	MDA-MB-231	7.7	55.9	>100	13.0	31.3	62.3	93.4	3.0	31.2	60.8	90.4	2.9	100.0	100.0	100.0	1.0	>100	>100	>100	1.0
	T-47D	2.8	6.5	10.2	3.6	22.8	61.8	>100	4.4	9.2	51.9	99.1	10.8	81.8	100.0	100.0	1.2	>100	>100	>100	1.0
**Pancreas**	AsPC-1	1.7	5.0	8.3	4.9	7.8	47.6	>100	13.1	7.2	39.2	80.4	11.2	58.6	100.0	100.0	1.7	65.7	>100	>100	1.5
	BxPC-3	2.0	5.6	9.3	4.7	12.8	53.1	93.4	7.3	22.1	55.2	88.3	4.0	81.5	100.0	100.0	1.2	>100	>100	>100	1.0
	MIAPaCa-2	3.1	5.9	8.7	2.8	28.8	58.5	88.1	3.1	4.5	6.9	9.3	2.1	45.8	71.1	96.4	2.1	>100	>100	>100	1.0
	PANC-1	4.0	7.0	6.3	1.6	•	•	•	•	22.6	52.9	83.3	3.7	•	•	•	•	•	•	•	•
**MEAN**		4.3	15.3	45.0	9.2	24.6	58.8	92.4	5.0	22.3	49.6	83.3	4.9	73.6	97.3	99.8	1.4	92.1	361.1	100.0	1.1

**Table 2:** The five substances with the highest correlation (PCC) to the selected sigma ligands as proposed by the COMPARE algorithm and their mechanism of action.

Ligand	PCC	Target Vector	Mechanism of Action (MoA)
**Siramesine**	0.601	dihydro-5-azacytidine	DNA damage [21]
	0.519	cyanomorpholino-ADR	Alkylating agent [22]
	0.507	cytembena	Inhibition of DNA and protein synthesis [23]
	0.487	anguidine	Inhibition of protein synthesis [21]
	0.462	caracemide	Inhibition of DNA synthesis [23]
**PB28 dihydrochloride**	0.724	pibenzimol hydrochloride	Inhibition of DNA replication [21]
	0.561	cytembena	Inhibition of DNA and protein synthesis [23]
	0.514	chloroquinoxaline sulfonamide	DNA damage [21]
	0.501	tamoxifen	Inhibition of DNA synthesis [23]
	0.46	6-mercaptopurine	Inhibition of DNA synthesis [23]
**Rimcazole dihychloride**	0.884	thalicarpine	Inhibition of DNA, RNA, and protein synthesis [24]
	0.687	spirogermanium	Inhibition of protein synthesis [25]
	0.541	caracemide	Inhibition of DNA synthesis [21]
	0.516	tetrocarcin A sodium salt	Inhibition of mitochondrial function [26]
	0.513	semustine (methyl-CCNU)	Alkylating agent [21]
**SM21 maleate**	0.585	anguidine	Inhibition of protein synthesis [21]
	0.523	caracemide	Inhibition of DNA synthesis [21]
	0.490	rhizoxin	Antimitotic agent [21]
	0.485	tetrocarcin A sodium salt	Inhibit mitochondrial function [26]
	0.466	didemnin B	Inhibition of protein synthesis [21]
**BD1047** **dihydrobromide**	0.618	spirogermanium	Inhibition of DNA, RNA, and protein synthesis [25]
	0.618	tamoxifen	Inhibition of DNA synthesis [21]
	0.575	chloroquinoxaline sulfonamide	DNA damage [21]
	0.566	thalicarpine	Inhibition of DNA, RNA, and protein synthesis [24]
	0.543	flavoneacetic acid	Change the permeability of the tumor vasculature [21]

## Data Availability

The data presented in this study are available within the article. For any further request contact the corresponding author.
